# Counseling interactions between patients living with persistent pain and pharmacists in Australia: are we on the same page?

**DOI:** 10.2147/JPR.S199017

**Published:** 2019-08-05

**Authors:** Esther TL Lau, Shirin H Tan, Yasmin J Antwertinger, Tony Hall, Lisa M Nissen

**Affiliations:** 1 School of Clinical Sciences, Queensland University of Technology, Brisbane, QLD, Australia; 2 School of Pharmacy, University of Queensland, Brisbane, QLD, Australia; 3 Clinical Research Center, Sarawak General Hospital, Kuching, Sarawak, Malaysia

**Keywords:** persistent, chronic, pain, pharmacist, counseling

## Abstract

**Background:**

People living with persistent pain in Australia often cannot access adequate care to manage their pain. Therefore, as the most accessible healthcare professionals, community pharmacists have an important role to play in helping to improve patient outcomes. Hence, it is important to investigate patient needs and expectations in terms of counseling interactions with pharmacists, along with pharmacists’ approach to counseling interactions with these patients.

**Method:**

The nature of patient–pharmacist counseling interactions was explored with seven patients (one focus group), and 10 practicing pharmacists (two focus groups, three semi-structured interviews). The themes identified informed the development of an online survey that was advertised online to patients and pharmacists across Australia.

**Results:**

A total of 95 patients and 208 pharmacists completed the survey. Overall, more than half of patients (77/95) were satisfied with the care provided by their pharmacist, but only a third (71/205) of pharmacists were satisfied with the care they provided to patients. The majority of patients (67/94) reported that pharmacists provided good information about medications. This aligned with pharmacists’ responses, as most reported focusing on medication side effects (118/188) and instructions for taking pain medication (93/183) during patient interactions. However, when asked about empathy and rapport from pharmacists, only half to two-thirds (48–61/95) of patients expressed positive views. Overall, half of the patients (39/75) wanted a caring, empathetic, respectful, and private conversation with the pharmacist, and nearly half (40/89) perceived the pharmacist's role as providing (new) information on alternative pharmacological and non-pharmacological therapies, including general advice on pain management.

**Conclusion:**

There was a disparity in the nature of the interaction and information that patients wanted from pharmacists, compared to what was provided by pharmacists. Training and education may help pharmacists to better engage in patient-centered care when interacting with people living with persistent pain, thereby improving health outcomes for these patients.

## Plain Language Summary

Inadequately managed pain is strongly associated with negative health outcomes for patients. Medicines are the most common treatment offered to people with persistent pain, but instead of being the solution, they can sometimes become the problem. People who take medicine for persistent pain may experience social stigmatisation and discriminationdue to a lack of understanding about the nature of persistent pain. Pharmacists can increase understanding of persistent painand improve patient outcomes by providing health information, and supporting people by being aware of their pain management needs. This study highlighted differences between the expectations and needs of people living with persistent pain, and the pharmacists' perceptions of the care and support required. That is, patients wanted pharmacists to be empathetic, to provide information on other treatments and general advice on pain management in addition to general medicines information. However, pharmacists focused on medicine side effects and how to take the medicine. Clearly pharmacists’ perception and understanding of pain management, needs to be realigned with patients needs. Unless change occurs, pharmacists may not provide the most adequate and appropriate care to patients living with persistent pain.

## Introduction

Persistent pain is Australia’s third most expensive health problem, costing the economy AU$48.3 billion annually.^1^ An estimated one in five Australians will experience persistent pain in their lifetime.^2^ Inadequately managed persistent pain has been associated with reduced quality of life, decreased work ability and productivity leading to early retirement, and increased mortality, as well as having links with depression and suicide. While up to 80% of people living with persistent pain could be effectively managed if they were able to access adequate care, statistics show that this care is accessible by less than 10% of patients.^1^–^4^

Access to adequate care in persistent pain management is limited by the number of sufficiently trained healthcare professionals.^2^ The paradigm of pain management is often based on acute pain, which usually resolves and disappears as tissue healing takes place, regardless of whether any interventions or medicines are used.^5^ On the other hand, pain becomes persistent (chronic) if the symptom persists beyond the duration when normal healing would be expected to occur, which is usually 3 months. Therefore, persistent pain is increasingly being recognized as being a disease in its own right rather than a symptom, and patients with persistent pain cannot expect their pain to disappear completely. Instead, management focuses on maintaining mobility and function, rather than a cure or complete absence of pain. New research about different pain phenotypes, e.g., nociplastic pain, is driving a shift in understanding and management of persistent pain.^6^ Furthermore, increasing opioid misuse and opioid-related deaths around the world are helping to bring these conversations into the light.^5^,^7^,^8^

Persistent pain is complex, and a multimodal approach is required, including considerations around biopsychosocial factors unique to each patient.^9^–^11^ Patients' self-efficacy in managing their pain condition is also influenced by their health literacy, with many patients viewing medicines as the panacea.^2^,^12^,^13^ Unfortunately, analgesic medicines, at best, reduce the pain experience by up to 50%,^5^ and the use of terminology such as “painkillers” leads to unrealistic patient expectations that they will achieve the complete absence of pain. The fact is that people with persistent pain conditions respond poorly to medicines, and may experience adverse effects associated with long-term use of analgesics.^11^,^14^,^15^

Pain is subjective and is whatever the experiencing person says it is, existing whenever the experiencing person says it does.^5^ Therefore, another commonly reported barrier to effective persistent pain management relates to the concept of the “sick role”. The sick role relates to the degree to which a person’s experience of illness is accepted by his or her surrounds, and is tied to the degree to which this illness experience is transformed into sickness, that is, the degree in which it becomes socially meaningful.^16^

Hence, when people do not fulfill the sick role, e.g., by not looking or appearing sick as per the stereotype, feelings of delegitimization and stigmatization can arise.^16^–^19^ In terms of persistent pain, this can manifest when others, including health professionals, doubt the reality or legitimacy of one’s pain, particularly when no objective signs of pain are visible.^16^–^19^

Pharmacists have an important role to play in supporting patients living with persistent pain, particularly where patients cannot easily access the multidisciplinary team needed to advise on the biopsychosocial aspects of pain.^2^,^20^ Pharmacist-led interventions of medication reviews,^21^ educational interventions,^22^ and pharmacist prescribing^23^ have reportedly resulted in statistically significant improvements in persistent pain management. For example, patients reported reductions in pain intensity, increased satisfaction, improved physical functioning, and reduced medication adverse effects. While pharmacists in Australia do not have prescribing rights as yet, they are currently able to implement all of the other aforementioned pharmacist-led interventions. Resources are also being allocated to investigate the role of community pharmacists in improving outcomes of patients with persistent pain, e.g., the Chronic Pain Medscheck Trial.^24^ Therefore, community pharmacists, as the most accessible healthcare professionals, can play a pivotal role in monitoring persistent pain and triaging acute pain.^15^,^25^,^26^ Pharmacists, as medicines experts, are well placed to provide information on the use of pharmacological (e.g., non-prescription, and complementary and alternative medications) and non-pharmacological interventions.^25^,^27^ Moreover, they can provide information on persistent pain, encourage timely referrals to relevant health services, and offer ongoing guidance and support for patients living with persistent pain, including those recently discharged from specialist services or pain clinics.^2^,^28^

Despite the patient–pharmacist counseling interaction being a fundamental role for pharmacists, it lacks recognition by physicians and patients and is often undervalued.^14^,^29^,^30^ There is also limited research exploring whether pharmacist counseling meets the needs and expectations of patients.^26^,^29^,^31^ Identifying and addressing any gaps in the patient–pharmacist counseling interaction can help to improve patient outcomes. It is therefore important to investigate the needs and expectations of patients living with persistent pain in terms of counseling interactions with pharmacists, along with pharmacists’ approach to counseling interactions with this cohort of patients.

## Materials and methods

### Study design

This was a mixed-methods study conducted in two phases. The first phase consisted of focus groups and semi-structured interviews, the results of which informed the development of the second phase, which was an online survey.^32^ The first phase ran from March to April 2011, and included two sets of focus groups/semi-structured interviews depending on participant availability. One set of focus groups/semi-structured interviews explored patients' opinions and experiences of their interactions with pharmacists, while the second set explored the perspectives of practicing pharmacists. Themes identified from the focus groups/semi-structured interviews informed the development of an online survey, which was advertised to patients and pharmacists in Australia between April and August 2011.

Ethics approval was obtained from the University of Queensland, School of Pharmacy Human Research Ethics Committee (approval number 2010/32). Each participant in the focus groups/semi-structured interviews provided written informed consent, while completion and submission of the online survey was taken as implied consent.

### Focus groups and semi-structured interviews

Using purposive snowball sampling recruitment, practicing pharmacists who had worked in a variety of practice settings were invited to participate through the research team’s personal contacts. Patients living with persistent pain were invited to participate through Chronic Pain Australia (CPA) and the Australian Pain Management Association (APMA). Immediately before each focus group/semi-structured interview, participants were asked to complete a short survey. The survey captured demographic information, and contained questions similar to the seeding questions used in the focus group/semi-structured interviews in the form of a five-point Likert scale with the anchors “strongly agree” to “strongly disagree”. These questions were to encourage responses that might be lost under the pressure of discussion with peers. Seeding questions (see Supplementary material) were used in the focus group/semi-structured interviews to facilitate discussion, maintain consistency, and reduce potential for interviewer bias. The focus groups and interviews were audio-recorded and transcribed verbatim.

### Survey development

The surveys were tailored to the pharmacist and patient respondent groups. The themes identified from the focus groups/semi-structured interviews were collated and used to develop the survey about the patient–pharmacist counseling interactions, in particular:

1. Patients' and pharmacists' perceptions and expectations of their interaction with each other
2. The role of a pharmacist in providing care for people living with persistent pain
3. Barriers and facilitators to pharmacists providing care

Demographic information was also collected, together with background data pertaining to the counseling provided by pharmacists. A combination of multiple-choice questions and five-point Likert scales with anchors “strongly disagree” to “strongly agree” was used. Respondents were also invited to share their views and opinions on how the counseling interaction between pharmacists and patients could be improved, in free-text open-ended questions. Surveys were piloted on a group of pharmacists for readability and understanding.

#### Participant recruitment

A link to the online version of the survey for patients with persistent pain was placed on the CPA website and emailed to APMA members, while a link to the survey for pharmacists was included in the Pharmaceutical Society of Australia e-bulletin, which was emailed to members nationwide.

### Statistical analysis

#### Focus groups/semi-structured interviews

The background and demographic information collected from the survey prior to the focus groups/semi-structured interviews was analysed using descriptive statistics (frequency counts, range and median). Transcripts from the focus groups/semi-structured interviews were manually coded and thematically analyzed to identify emergent themes.

#### Survey

Cronbach’s alpha coefficient was used to evaluate the internal consistency reliability of the five-point Likert scales. The alpha values for the pharmacist and patient groups were calculated separately using SPSS 21.0 software (IBM Corp., Armonk, NY, USA), with nine and 10 questions included in the calculation, respectively. The five-point Likert scale was collapsed into a three-point scale: agree/positive (agree, strongly agree), impartial/neutral, and disagree/negative responses (disagree, strongly disagree), to analyze the respondents’ level of agreement with the statements for reporting purposes. The remainder of the questions were analyzed using descriptive statistics (frequency counts, range and median), while the responses to the free-text open-ended questions were manually coded and analyzed to identify emergent themes. Some pharmacists and patients did not answer all questions, so the response for each question was calculated based on the actual number of respondents.

## Results

### Focus groups/semi-structured interviews

#### People living with persistent pain

##### Demographics

All seven patients with persistent pain took part in one focus group. The pain types reported were: persistent postoperative pain (2/7), persistent pain following multiple fractures (1/7), neuropathic pain (1/7), complex regional pain syndrome (1/7), and unknown or undiagnosed pain (2/7). Most of the people with persistent pain were female (5/7), aged 50–90 years (median 56 years), and had experienced persistent pain for 4–29 years (median 9 years).

Almost all patients would speak to their doctor if they had concerns about their pain or pain medications, (6/7), followed by the pharmacist (4/7), physiotherapist (2/7), support group members (1/7), and family members (1/7). Similarly, when seeking information about pain or medications, they would obtain information from their doctors (7/7), pharmacists (6/7), the internet (2/7), and their physiotherapists (1/7). Almost all of the patient participants (6/7) were comfortable talking about their condition and medications with a pharmacist, were confident about managing their medications after listening to the pharmacist, and felt that the pharmacist understood when they spoke about their condition. The remaining participant expressed an impartial or neutral view.

##### Pre-focus group/semi-structured interview survey

In the pre-focus group/semi-structured interview survey, when starting a new medication, the majority of the patients reported that pharmacists spent 1–5 minutes (4/7) speaking to them about new pain medication, which then reduced to <1 minute (5/6) with repeat medications. Patients wanted and expected pharmacists to help with medicines information, e.g., how to use the medicine, side effects, and contraindications. They also expected the opportunity to have questions about their pain and medicines answered, and to be followed up if any changes in their care occurred. A holistic and patient-centered approach to pain management was also wanted, i.e., incorporating non-pharmacological treatment options, the ability to discuss issues in private counseling areas, and to be believed when they spoke about side effects.

##### Patient perceptions of their interactions with pharmacists

In terms of the overall experience and interactions with the pharmacist, patients reported different experiences. While some noted negative interactions with their pharmacist, others were comfortable about approaching their pharmacist with questions.

“I think one of the biggest problems is they [the pharmacist] don’t listen [to the person with the pain]. It was a matter of they know more because they’ve been educated, but that’s my body, I know more.”

“He [the pharmacist] takes a lot of time with people and I guess I do feel like I can ask him questions, whether they are silly questions or not, if it is something a bit concerning, I know I can ask him.”

##### The role of a pharmacist in providing care for patients with persistent pain

Similar themes were generated during the focus group discussions, in that patients perceived the role of the pharmacist to be the medicines expert, and relied on them to help with answering questions about medicines.

“… pharmacists are the medication experts and so … that’s who I expect to be able to go to if I’ve medication sort of questions.”

##### Barriers and facilitators to pharmacists providing care

Some patients also indicated that the pharmacist was a valuable source of information for understanding about, and management of, their pain condition. However, it was evident that the manner in which this information was presented, collated, and explained was extremely important in terms of patient receptivity and rapport building.

“Pharmacists have to be careful too when just pushing paper out to patients. It’s very easy to just print them out and shove them to the patients.”

“I had to change one medication that I was on and he [the pharmacist] printed out all the stuff from the pharmaceutical company. He monitored what I was getting … and made sure I understood too.”

Other recommendations suggested by patients in terms of the role that pharmacists could play in the management of persistent pain were to increase awareness and understanding of persistent pain and its management, and to promote how pharmacists could help with medicines.

“I don’t think I was aware that pharmacists know more about medications than the doctors so I think that’s something the pharmacists should let the community be aware of. Promote it.”

#### Pharmacists

##### Demographics

Two focus groups (n=3, n=4) and three semi-structured interviews were conducted with the pharmacists. Most of the pharmacists were females (9/10), aged 25–60 years of age (median 0 years), and had practiced as pharmacists for 2–40 years (median 10 years), with many working across several fields of practice in community pharmacy (n=7), hospital pharmacy (n=5), research (n=4), academia (n=2), and government (n=1).

##### Pre-focus group/semi-structured interview survey

In the pre-focus group survey, most pharmacists indicated that they spent 5–10 minutes speaking about new pain medications (4/9) and 1–5 minutes on repeat medications (7/10). Most participants believed that their role was to provide information on medication (9/10), advise on side effects and their management (7/10), review pain control (5/10), and provide treatment alternatives (2/10). Some other less common responses were providing reassurance, detecting drug interactions, and providing the consumer medicines information leaflet. All of the pharmacist participants were comfortable and confident about counseling people with persistent pain, but many (7/10) were hesitant about dispensing large amounts of opioids to patients with persistent pain, and the remainder held an impartial or neutral viewpoint. The majority were also comfortable listening to patients talk about their pain and medications (9/10), with 1/10 expressing an impartial or neutral opinion. Similarly, 9/10 believed that every persistent pain patient is unique, with one respondent disagreeing with that view.

##### Pharmacists' perceptions of their interactions with patients

Similarly to the patient respondents, some pharmacists described positive interactions with patients, while others found patients to be defensive and non-receptive.

“So a patient’s or a customer’s perception of what value are they gonna get from that interaction. So if they assume they are not gonna get, so “Look, I go to my specialist for help with my pain, what do you know?” And maybe it’s from past experience that they haven’t really got appropriate advice or condescending, or restricted supply without really knowing why.”

“… even if you ask open-ended questions, they give you closed answers like “yes” and “no”, then it’s very difficult to take the conversation any further. If they are receptive, then you can go further into it.”

##### The role of a pharmacist in providing care for patients with persistent pain

Pharmacists also noted differences in patient health literacy and self-efficacy to manage their pain and pain medications, which would then influence what pharmacists perceived their role to be in providing care and information for patients with persistent pain.

“I actually did find most of them are quite a unique group of people in that they get on internet and find information about their drugs, what’s available for them, what they can have and that sort of stuff.”

“The number of patients who are confused or have misunderstanding about paracetamol and its many other preparations and the role of paracetamol continues to astonish me. That is something simple that you can do.”

##### Barriers and facilitators to pharmacists providing care

Some respondents recognized that many pharmacists lacked empathy when interacting with patients with persistent pain, and the potential/perceived stigma that these patients faced when doubted about the genuineness of their pain condition.

“I don’t think I ever give the poor little old lady with arthritis sufficient empathy because I hadn’t experienced it, I couldn’t appreciate it so perhaps we really need to train young pharmacists in the school, about pain but then, at that age, are we really being receptive?”

“… they [persistent pain patients] will say “They just think I’m drug addict, they just think I’m drug seeking or seeking opioids, they don’t realize I’m in pain.” So there is still this stigma associated with being on opioids in the community …”

Pharmacists' recommendations on optimizing interactions with patients living with persistent pain included more education and specialized training for pharmacists; ability to make referrals; building empathy, relationships, and rapport with patients; and ensuring adequate resources to allow meaningful interactions.

“Those short interactions could definitely be improved unbelievably so especially with the referral to other healthcare providers whether to a multidisciplinary pain clinic or having a bit more understanding of what their conditions are.”

“You have that knowledge … but it is the way you delivering that actually gives any benefit to the patient.”

“And time constraints, in the community pharmacy, if you’re the only pharmacist and they don’t have enough technicians to help you … and there’s no way I can really have a good conversation with them, absolutely not.”

### Survey

#### Demographics

A total of 208 pharmacists and 95 people with persistent pain completed the survey. Survey responses were reliable in both the pharmacist and patient surveys (Cronbach’s alpha =0.949 and 0.794, respectively). The majority (79/93) of the patient respondents were female, with just over half (50/95) aged between 41 and 60 years (median age range =41–50 years). Approximately half of the people with persistent pain (43/84) had lived with persistent pain for 1–10 years, with the median being 6–10 years (Table 1). The ages of the pharmacist respondents were more evenly distributed, with the majority being female (140/204). Most of the pharmacists worked in community pharmacy, but representation from a range of backgrounds was obtained (Table 1).

**Table 1:** Demographics of survey participants

Characteristics	Survey participants	
	Patients with persistent pain	Pharmacists
Gender		
Male	14/93 (15.1%)	64/204 (31.4%)
Female	79/93 (84.9%)	140/204 (68.6%)
Age (years)		
≤30	17/95 (17.9%)	56/204 (27.5%)
31–40	15/95 (15.8%)	34/204 (16.7%)
41–50	25/95 (26.3%)	40/204 (19.6%)
51–60	25/95 (26.3%)	37/204 (18.1%)
≥61	13/95 (13.8%)	37/204 (18.1%)
Years experiencing persistent pain		
<1	4/84 (4.8%)	–
1–5	23/84 (27.4%)	–
6–10	20/84 (23.8%)	–
11–15	13/84 (15.5%)	–
16–20	10/84 (11.9%)	–
21–25	5/84 (6.0%)	–
26–30	6/84 (7.1%)	–
≥31	3/84 (3.6%)	–
Years in profession		
≤5	–	50/206 (24.6%)
6–10	–	24/206 (11.8%)
11–15	–	13/206 (6.4%)
16–20	–	16/206 (7.9%)
21–25	–	13/206 (6.4%)
26–30	–	29/206 (14.3%)
31–35	–	19/206 (9.4%)
36–40	–	7/206 (3.4%)
41–45	–	20/206 (9.9%)
46–50	–	6/206 (3.0%)
≥51	–	6/206 (3.0%)
Area of practice (some pharmacists reported working in more than one area of practice)		
Academia/research	–	4/205 (2.0%)
Accredited pharmacist	–	18/205 (8.8%)
Community	–	157/205 (76.6%)
Government	–	1/205 (0.5%)
Hospital	–	21/205 (10.2%)
Industry	–	1/205 (0.5%)
Military	–	1/205 (0.5%)
Professional organization/association	–	2/205 (1.0%)

#### Patients' perceptions of their interactions with pharmacists

All patients spoke to their doctor (general practitioner [GP] and/or specialist) about concerns with their pain or medications, of whom approximately half (45/95) would speak to a pharmacist in combination with their doctor. However, none would speak to the pharmacist only, and 20/95 would seek information from other sources. These included combinations of complementary and alternative medications or therapies, eg, traditional Chinese medicines; massage therapists; allied health professionals, including physiotherapists and psychologists; pain clinics; and the internet or telephone help lines.

In terms of seeking information about their pain or medications, the most popular source of information was the doctor (GP and/or specialists) (81/95), followed by the internet (65/95), then the pharmacist (59/65). Overall, 19/95 of patients sought information from “other” sources, these being allied health professionals, support groups, complementary and alternative therapists, books, patient medicines information leaflets, and the National Prescribing Service’s Medicine hotline.

Almost all patient respondents (89/95) agreed that pharmacists knew a lot about medications (Table 2). The majority held positive views about the pharmacist being available to help them with their concerns, and were comfortable speaking to the pharmacist about their pain and medications (n=66–80). Patients perceived pharmacists to provide good information about medications (67/94), but were less positive about the information received about the pain condition (34/95). While many were confident about how to take their medications after speaking to the pharmacist (77/95), fewer were satisfied with the amount of care provided by the pharmacist (55/95). When patients were asked questions relating to empathy and rapport, only half to two-thirds (48–61/95) held positive views (Table 2).

**Table 2:** Patients' survey responses

Questions	Strongly disagree	Disagree	Neutral	Agree	Strongly agree	Number of respondents
I believe pharmacists know a lot about medicines	1 (1.1%)	1 (1.1%)	4 (4.2%)	44 (46.3%)	45 (47.4%)	95/95
The pharmacist provides me with good information about my pain	6 (6.3%)	21 (22.1%)	34 (35.8%)	20 (21.1%)	14 (14.7%)	95/95
The pharmacist provides me with good information about my medications	3 (3.2%)	10 (10.6%)	14 (14.9%)	43 (45.7%)	24 (25.5%)	94/95
I feel that the pharmacist is always available to help me with my concerns	6 (6.4%)	8 (8.5%)	14 (14.9%)	37 (39.4%)	29 (30.9%)	94/95
I prefer to talk to the pharmacist in a private counselling area	7 (7.4%)	6 (6.3%)	30 (31.6%)	33 (34.7%)	19 (20.0%)	95/95
I feel the pharmacist cares about me	5 (5.3%)	10 (10.5%)	32 (33.7%)	31 (32.6%)	17 (17.9%)	95/95
I feel the pharmacist is willing to listen to me	2 (2.1%)	8 (8.4%)	27 (28.4%)	35 (36.8%)	23 (24.2%)	95/95
I feel comfortable talking about my condition with a pharmacist	2 (2.1%)	5 (5.3%)	16 (16.8%)	43 (45.3%)	29 (30.5%)	95/95
I feel comfortable talking about my medications with a pharmacist	1 (1.1%)	5 (5.4%)	7 (7.5%)	44 (47.3%)	36 (38.7%)	93/95
I feel the pharmacist believes me when I talk about my condition	2 (2.1%)	9 (9.5%)	23 (24.2%)	33 (34.7%)	28 (29.5%)	95/95
I am satisfied with the amount of care the pharmacist provides	5 (5.3%)	8 (8.4%)	27 (28.4%)	31 (32.6%)	24 (25.3%)	95/95
I feel confident about how to take my medications after listening to the pharmacist	2 (2.1%)	4 (4.2%)	12 (12.6%)	44 (46.3%)	33 (34.7%)	95/95

#### Pharmacists' perceptions of their interactions with patients

The majority of pharmacists were comfortable listening to people speak about their pain condition (179/206), and were comfortable and confident about providing counseling to patients with persistent pain (164–176/207) (Table 3). However, there were mixed views about the needs of patients with persistent pain, with 92/206 pharmacists believing that patients all had similar issues; and 74/208 indicated that they had difficulty identifying genuine patients with persistent pain. Overall, only 71/205 pharmacists were satisfied with the amount of care they provided to patients (Table 3).

**Table 3:** Pharmacists' survey responses

Questions	Strongly disagree	Disagree	Neutral	Agree	Strongly agree	Number of respondents
I feel comfortable when counselling patients with chronic pain	0 (0%)	14 (6.8%)	17 (8.2%)	132 (63.8%)	44 (21.3%)	207/208
I feel confident when counselling patients with chronic pain	0 (0%)	20 (9.7%)	23 (11.1%)	125 (60.4%)	39 (18.8%)	207/208
I feel comfortable listening to patients with chronic pain talk about their pain	0 (0%)	8 (3.9%)	19 (9.2%)	123 (59.7%)	56 (27.2%)	206/208
I believe that patients with chronic pain have similar issues	5 (2.4%)	48 (23.3%)	61 (29.6%)	77 (37.4%)	15 (7.3%)	206/208
I believe I am able to confidently deal with complex issues and experiences faced by patients with chronic pain	3 (1.5%)	56 (27.2%)	53 (25.7%)	80 (38.8%)	14 (6.8%)	206/208
I feel that I am able to spend sufficient time counselling patients with chronic pain	15 (7.2%)	71 (34.3%)	43 (20.8%)	62 (30.0%)	16 (7.7%)	207/208
I am satisfied with the amount of care I provide to patients with chronic pain	7 (3.4%)	62 (30.2%)	65 (31.7%)	59 (28.8%)	12 (5.9%)	205/208
I sometimes feel hesitant about dispensing large amounts of opioids to patients with chronic pain	9 (4.4%)	44 (21.4%)	36 (17.5%)	90 (43.7%)	27 (13.1%)	206/208
I have difficulty in identifying genuine pain patients	6 (2.9%)	66 (32.4%)	58 (28.4%)	67 (32.8%)	7 (3.4%)	204/208
I am happy for other pharmacy staff (eg, pharmacy assistants) to provide counselling for patients with chronic pain	57 (27.7%)	104 (50.5%)	31 (15.0%)	12 (5.8%)	2 (1.0%)	206/208
I would like to talk to patients with chronic pain in a private counselling area/room	4 (2.0%)	18 (8.8%)	57 (27.8%)	89 (43.4%)	37 (18.0%)	205/208

#### The role of a pharmacist in providing care for patients with persistent pain

Patients were most frequently concerned about side effects from their medications (33/89), which is what pharmacists thought they focused on during their interactions (118/188) (Table 4). However, patients reported that the most typical conversation they had with their pharmacist was about how to take the medication (41/90), and this was what pharmacists most commonly perceived their role to be when caring for patients living with persistent pain (93/183). However, what patients wanted was a caring, empathetic, respectful, and private conversation with the pharmacist (39/75). The role that patients saw for pharmacists was to inform them about alternative pharmacological and non-pharmacological therapies, and especially new information (40/89) (Table 4).

**Table 4:** Patients’ concerns (ordered by frequency) about their pain and medications, what they want from their interaction with pharmacists, conversation that takes place during a typical interaction with the pharmacist, and the perceived role of pharmacists in managing pain and medications; and pharmacists’ focus (ordered by frequency) when interacting with patients with persistent pain, and self-perceived role in the management of people with persistent pain

Patients (n=89): Concern(s) patients have about their pain and medications		Patients (n=75): What patients want out of their interaction with pharmacists		Patients (n=90): What patients and their pharmacist talk about in a typical interaction		Patients (n=89): What patients think a pharmacist can help with in managing pain and medications		Pharmacists (n=188): What pharmacists focus on in their interaction with patients with persistent pain		Pharmacists (n=183): Roles pharmacists perceive they can play in the management of a patient with persistent pain	
Medication side effects	33.7%	Enough time for an empathic, caring, friendly, respectful, private conversation with the pharmacist	52.0%	Information about their medications, including instructions on how to take the medication	45.6%	Suggesting adjuvant and alternative pharmacological and non-pharmacological options (especially new information)	44.9%	Medication side effects	62.8%	Information about their medications, including instructions on how to take the medication	50.8%
Lack of effective pain control	32.6%	Suggesting adjuvant and alternative pharmacological and non-pharmacological options (especially new information)	36.0%	Do not interact with the pharmacist much; only have very brief interactions with the pharmacist	31.1%	Showing empathy and building rapport	30.3%	How to manage the medications – persistent pain management	50.0%	How to manage the medications – persistent pain management	45.4%
Drug interactions	16.9%	Information about their medications, including instructions on how to take the medication	33.3%	Medication side effects	28.9%	How to manage the medications – persistent pain management	22.5%	Suggesting adjuvant and alternative pharmacological and non-pharmacological options	42.0%	Suggesting adjuvant and alternative pharmacological and non-pharmacological options	31.7%
Stigma	14.6%	Medication side effects	14.7%	Non-health related: general conversations, building rapport	15.6%	1. Medication side effects AND 2. Information about their medications, including instructions on how to take the medication	18.0%	Information about their medications, including instructions on how to take the medication	30.3%	Medication side effects	30.6%
Addiction/dependence	12.4%	1. Drug interactions AND 2. Having the pharmacist check their understanding of medications and pain management	12.0%	Drug interactions	13.3%	Drug interactions	14.6%	Checking if they are achieving adequate pain control	16.5%	Addressing and providing general support regarding general concerns	20.2%

**Figure 1:**
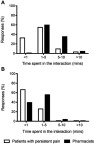
Amount of time spent talking in a typical interaction, as reported by people with persistent pain and pharmacists, for (**A**) new medication (n=75 and n=205, respectively), and (**B**) repeat medication (n=73 and n=203, respectively).

The general consensus between patients and pharmacists was that between 1 and 5 minutes were spent talking about new pain medications (Figure 1). However, patients most frequently reported pharmacists spending <1 minute counseling on repeat pain medications, while pharmacists most frequently reported spending 1–5 minutes. The overall rightward skew in the pharmacists’ responses indicated that they perceived having spent more time counseling patients about their medications, compared to what the patients reported (Figure 1).

#### Barriers and facilitators to pharmacists providing care

The most common barrier identified by pharmacists was that patients harbored negative feelings and were defensive or non-receptive to interactions, and the strategy offered to overcome this was to build rapport, communicate, and show empathy for patients (Table 5). A little over half of the patients (52/95) and pharmacists (126/205) preferred the counseling interaction to take place in a private area of the pharmacy (Tables 2 and 3). While the majority of pharmacists (161/206) did not want pharmacy staff who were not pharmacists to provide counseling to people with persistent pain, only 78/207 pharmacists felt that they were able to spend sufficient time counseling patients with persistent pain.

**Table 5:** Barriers and proposed facilitators (ordered by frequency) identified by pharmacists when counseling people with persistent pain (n=168)

Barriers		Facilitators	
Patients’ negative feelings (eg, toward their medications, healthcare professionals, frustration with lack of pain relief from medication), defensiveness or non-receptivity to interactions	38.1%	Build rapport, communicate, show empathy for patients	23.2%
Insufficient time and/or lack of privacy or appropriate area for counseling	37.5%	Educate patients about their medications, pain management	20.2%
Patients’ lack of knowledge about medication management, negative feelings and stigma about drug tolerance/dependence/addiction	25.6%	Provide a private area for interaction	8.9%
Pharmacists’ doubts about patient genuineness, lack of patient honesty, and concern about drug abuse/dependence/addiction/overuse	16.7%	Collaborate and share information/feedback with other healthcare professionals involved	8.3%
Pharmacists’ lack of knowledge about managing patients with chronic pain	8.9%	Education for pharmacists/healthcare professionals	7.1%
Pharmacists are not informed of pain management protocol/plan to ensure that consistent information and advice is provided	7.7%	Monitor medication usage/dispensing, staged supply of medications	5.4%
Pharmacists lack background information about patients’ condition/medication history	7.1%	– Ensure sufficient staff rostered to work – Recommend home medicines reviews (HMRs) – Provide printed or better resources for counseling/interaction	4.8%
Pharmacists lack empathy and compassion	6.5%	Remuneration for counseling	4.2%
Patients do not recognize pharmacists’ roles	6.0%		
Barriers to communication, eg, non-English speaking	4.8%		
Other comorbidities are overlooked/not managed adequately (eg, depression)	4.2%		
Patients are unable to access resources, eg, pain clinics – issues around waiting times and locality	3.0%		
Doctors are not prescribing in accordance with most up-to-date evidence/guidelines	1.8%		
Lack of remuneration for pharmacists for counseling	1.2%		

## Discussion

### Patients’ perceptions of their interactions with pharmacists

Overall, patients indicated that pharmacists could improve in their interactions with patients. Patients generally held positive views about the medication information provided by pharmacists, but were less satisfied with the empathy and rapport aspect of the interaction (Table 2). Therefore, the way in which information was delivered, i.e., the empathy and rapport aspects of the interaction, was at least as important as the delivery of the actual information per se. This is consistent with research showing patient satisfaction being significantly correlated with empathetic skills of physicians, i.e., a friendly manner and respect for patients' feelings.^33^ More specifically, adopting a patient-centered approach, e.g., demonstrating empathy and encouraging shared decision-making, has been shown to improve the management of patients with persistent pain.^34^ The desire for empathy can arise from feelings of stigmatization and delegitimization, which are commonly reported by people with persistent pain, particularly when there is a lack of diagnosis or objective physical sign that pain is present.^16^,^18^,^19^ Patients also often report negative experiences with health professionals, and describe feelings of being rejected, ignored, belittled, and not being believed or taken seriously, as they are met with skepticism and a lack of understanding from health professionals.^17^–^19^ Pharmacists and all healthcare professionals have an important role to play in reducing the patient’s feelings of stigmatization or estrangement by showing empathy and building rapport with patients,^18^ which improves patient satisfaction and increases adherence to treatment.^35^

### Pharmacists' perceptions of their interactions with patients

The pharmacist survey responses suggest a gap in knowledge, since almost half of the pharmacist respondents believed that all patients with persistent pain had similar issues (Table 3). Persistent pain is a complex condition, sometimes with no known underlying cause, and each patient’s pain experience is unique.^2^ The pain management paradigm is most commonly based on the experience of acute pain, and unless educated otherwise, pharmacists will not be able to provide adequate and appropriate care to patients living with persistent pain.^4^,^18^ Similarly, pharmacists’ attempts to verify whether a patient's pain experience is “genuine” can further perpetuate patient feelings of stigmatization and delegitimization, and the perception of pharmacists lacking empathy (Table 3). This then reinforces pharmacists’ perceptions of patients being defensive and non-receptive. This lack of understanding about persistent pain and its management is not unique to Australia or to pharmacists. In fact, society at large, including many healthcare professionals, has a poor understanding of persistent pain and its management.^2^ Upskilling healthcare professionals is the first step toward increasing awareness of persistent pain and its management to help reduce stigmatization more broadly, and toward improving the management, outcomes, and experiences of patients living with persistent pain.

### The role of a pharmacist in providing care for patients with persistent pain

Patients and pharmacists held different views about the pharmacist's role in assisting people with persistent pain. There were also differences between the perceptions of patients and pharmacists in terms of what was discussed during a typical interaction. While pharmacists thought they were focusing on medication side effects, which was what most patients were concerned about, what patients took away from the interaction was general medicines information, including instructions on how to take the medication. Similarly, patients thought that a pharmacist could help with their pain management by providing information about a holistic approach to pain management, particularly new information about pharmacological and non-pharmacological options, and emphasized wanting sufficient time and empathy from the pharmacist (Table 4).

Not unexpectedly, much of what patients wanted was relevant and personalized information regarding their medications.^26^ The concerns reported by patients were also consistent with those commonly reported in literature, e.g., fear of side effects and addiction to pain medications, stigma, loss of control, frustration with health professionals, the lack of effective treatments, not being believed, and perceived lack of empathy.^16^,^18^,^19^,^36^ However, more work is required to better understand interactions between pharmacists and patients, as most studies focused on the information that pharmacists provided, rather than the perception of the patients involved.^31^

As well as differing perspectives on the contents and nature of the interaction, there were apparent differences between the amounts of time perceived to be spent during the counseling interactions (Figure 1). Pharmacists tended to overreport time spent with patients and the number of patients counseled on a day. This was especially true of repeat prescriptions, potentially due to beliefs held by pharmacists that patients with persistent pain knew a lot about their medicines and a perceived lack of interest from patients.^37^

This breakdown in communication could contribute to the lack of satisfaction with care given and received by pharmacists and patients, respectively, and also contribute to an increased burden of persistent pain for individuals.^4^,^38^,^39^ It may also explain why pharmacists were only the third source of contact/information for patients who had concerns or were seeking information about their pain medications and pain condition. This is despite pharmacists being the most accessible healthcare professionals and patients recognizing that pharmacists knew a lot about medicines. Not unexpectedly, the doctor was the preferred point of contact for patients because the doctor has the ability to make changes to the care plan or medications where necessary. However, patients preferred using the internet – ostensibly for convenience – before pharmacists. They would also seek information about medications from healthcare providers who had little or no training about medications. This suggests that pharmacists could be lacking in their patient-centered approach to care, and need to be more assertive in building rapport and being more responsive to patients’ needs. All healthcare professionals also play an important role in informing patients about how to interpret and select reliable information from the internet, particularly since low levels of health literacy have been linked to poor health outcomes for patients with persistent pain.^40^

### Barriers and facilitators to pharmacists providing care

The most common barriers identified by pharmacists were patients’ negative feelings, defensiveness, and non-receptivity to interactions. This is akin to the dissatisfaction voiced by patients with regard to the lack of empathy and rapport encountered during their interactions with pharmacists. It is well documented that fear of being stigmatized for taking opioids, concerns around pain management, and fear of addiction can cause patients to become defensive and unreceptive to pharmacist counseling.^18^,^35^,^41^ As well as decreasing the effectiveness of medication counseling interactions with pharmacists, these patient barriers can further complicate pain assessments and treatments when interacting with other healthcare professionals.^41^

The most commonly identified facilitator was therefore to build rapport, communicate, and show empathy for patients, as preconceived beliefs and previous experiences can become barriers to effective pharmacist counseling.^31^ As the pain management paradigm is most commonly based on the experience of acute pain, these beliefs and concerns from pharmacists can be minimized through specialized knowledge and training, which would better facilitate empathic interactions with patients.^31^

Pharmacists reported a lack of time and privacy as another major barrier to counseling. Many pharmacists did not have enough time to spend with patients, and patients also reported that they typically had little or no interaction with the pharmacist. Despite this lack of time and interaction, the majority of pharmacists did not want other pharmacy staff (who were not pharmacists) to provide counseling for patients with persistent pain, suggesting that pharmacists had some awareness of the complexities associated with the management of persistent pain. Therefore, in addition to increasing the knowledge of healthcare professionals, adequate resourcing is required to ensure that appropriately trained staff are able to spend sufficient time to have meaningful counseling interactions with patients living with persistent pain.

### Limitations

The main limitation of this study is the small sample size, which was related to the channels available to be used for recruitment at the time of the study. Similarly, a sampling bias could have occurred as only interested participants would have voluntarily completed in the survey, and it was not possible to ensure or verify the identity of the respondents. Also, while these data were originally collected in 2011, and only Australian patients and pharmacists were invited to participate, it is unfortunate that the findings are still relevant and are still issues that have yet to be addressed for patients.^1^ Furthermore, the most recent publications on persistent pain management more broadly would suggest that the issues identified in the study are not unique to Australia.^42^–^44^ In addition, only face validity was established in this study, so future research could investigate validating this survey, and also including more negatively phrased questions to ensure that participants are not subconsciously led to agree on the questions. Nevertheless, this research serves as an exploratory study of the issues surrounding pharmacists and their interactions with patients living with persistent pain, and highlights the need for more research in this area.

## Conclusion

In highlighting the gaps between expectations and needs of patients with persistent pain, and the approach and focus of pharmacists’ attention, it is apparent that a realignment in the understanding of persistent pain is required. Pharmacists focus on the provision of medication information, while patients with persistent pain would like information on other treatments and general advice on pain management. Inadequate knowledge by pharmacists with regard to persistent pain leads to pharmacists’ low level of confidence in counseling patients and a perceived lack of empathy from patients with persistent pain. Further education and training for pharmacists may help to improve their care and management of these patients. Similarly, empathy and patient-centered care need to be incorporated into pharmacists’ interactions with patients with persistent pain, in order to build rapport and trust relationships with patients and improve patient outcomes.

## Supplementary material

Focus group seeding questions/interview guide

Patients living with persistent pain:

1. Can you tell me about your chronic pain? How is your pain managed?
2. How do health professionals help you with your pain and medications?
3. In terms of your medicines, what do you need help with?
4. Who do you usually go to for help or information? Why?
5. Tell me about what happens when you go to a pharmacy. Does the service differ from one pharmacy to the other?
6. What do you think about the interaction that you experienced before? What do you think was good about it? Is there anything else that you think the pharmacist can help you with?

Community pharmacists:

1. Can you describe a typical counselling session you’ve had with a chronic/persistent pain patient? ie, environment, duration, content
2. Can you describe the nature of your relationships with patients with chronic/persistent pain?
3. How do you think you can contribute to chronic pain patients’ management of their condition? How often do you do this? How well is it accepted by the patients?
4. What do you usually focus on when counselling a chronic/persistent pain patient? Are there any issues about counselling chronic pain patients that you encounter/are worried about? ie, communication (level of understanding), dependence issue, authenticity of prescriptions, need for opioids, time constraint, etc
5. Is there any improvement or change that you think will be useful in optimising a counselling session (with a chronic/persistent pain patient)?
